# A disinhibition-based model of CA1 place field sequences

**DOI:** 10.64898/2026.10.08.757765

**Published:** 2026-10-15

**Authors:** Laura Green, Mihály Vöröslakos, György Buzsáki, John Rinzel

## Abstract

Because of the paucity of pattern-generating recurrent excitation in the CA1 hippocampal region, place field sequences are assumed to be inherited from upstream regions. Alternatively, disinhibition may play a role in shaping pyramidal cell activity. We propose a model of CA1 activity using one layer of excitatory cells and one layer of inhibitory cells, with local excitatory- to-inhibitory connectivity, broad inhibitory-to-excitatory connectivity without recurrent excitatory connections, and broad inhibitory-inhibitory connections. The layers need not follow a topographic organization; instead they can represent sequences of place cells structured by local inhibition. Our mathematical model of CA1 neurons with data-inspired connectivity demonstrates place fields sharpened by disinhibition, mimicking a recently discovered decrease in inhibitory input within place fields and matching in vivo patterns of disinhibition. Mathematical analysis of our model suggests the importance of inhibitory-to-excitatory connections in control of place cell firing rate and sparsity. We also demonstrate that a disinhibition-based mechanism of pattern generation is more flexible without recurrent excitation than the recurrent excitation-rich CA3-like model. Finally, we demonstrate disinhibition as an efficient mechanism for internally-generated sequential activity, where the speed of sequential change depends on both input strength and inhibitory-to-excitatory connection strength. Overall, our model suggests that theta time-scale disinhibition is a foundational mechanism of self-organized sequential activity in the CA1 region, and, possibly, in other networks.

**Author Summary:**

The hippocampus is thought to be important for learning and memory. The main output of the hippocampus is through the CA1 region, which contains cells that demonstrate spatially-informative firing patterns. Previous models of place cell activity include recurrent excitatory or increases of inhibitory input within a place field, though it is well known that CA1 exhibits only weak connections between excitatory cells. We provide a population model of CA1 place cell activity without recurrent excitation that replicates many characteristics of place cell activity, such as a decrease in inhibitory input within a place field and increasingly sparse activity with familiarity in the environment. We also demonstrate intrinsic sequential activity through disinhibition. We propose that disinhibition dominates theta-timescale activity in the CA1 region of the hippocampus.

## Full Text Availability

The license terms selected by the author(s) for this preprint version do not permit archiving in PMC. The full text is available from the preprint server.

